# Genetic population structure of the malaria vector *Anopheles baimaii *in north-east India using mitochondrial DNA

**DOI:** 10.1186/1475-2875-11-76

**Published:** 2012-03-20

**Authors:** Devojit K Sarma, Anil Prakash, Samantha M O'Loughlin, Dibya R Bhattacharyya, Pradumnya K Mohapatra, Kanta Bhattacharjee, Kanika Das, Sweta Singh, Nilanju P Sarma, Gias U Ahmed, Catherine Walton, Jagadish Mahanta

**Affiliations:** 1 Regional Medical Research Centre, NE (ICMR), Dibrugarh-786001, Assam, India; 2 Department of Ecology and Evolution, Imperial College London, Silwood Park SL5 7PY, UK; 3 College of Veterinary Sciences, Assam Agriculture University, Guwahati-781022 Assam, India; 4 Assam Agricultural University, Jorhat - 785013 Assam, India; 5 Department of Biotechnology, Gauhati University, Guwahati-781014 Assam, India; 6 Faculty of Life Sciences, University of Manchester, Oxford Road, Manchester M13 9PT, UK

**Keywords:** *Anopheles baimaii*, Cytochrome oxidase II, Southeast Asia, Malaria vector, North-east India, Population genetics

## Abstract

**Background:**

*Anopheles baimaii *is a primary vector of human malaria in the forest settings of Southeast Asia including the north-eastern region of India. Here, the genetic population structure and the basic population genetic parameters of *An. baimaii *in north-east India were estimated using DNA sequences of the mitochondrial cytochrome oxidase sub unit II (COII) gene.

**Methods:**

*Anopheles baimaii *were collected from 26 geo-referenced locations across the seven north-east Indian states and the COII gene was sequenced from 176 individuals across these sites. Fifty-seven COII sequences of *An. baimaii *from six locations in Bangladesh, Myanmar and Thailand from a previous study were added to this dataset. Altogether, 233 sequences were grouped into eight population groups, to facilitate analyses of genetic diversity, population structure and population history.

**Results:**

A star-shaped median joining haplotype network, unimodal mismatch distribution and significantly negative neutrality tests indicated population expansion in *An. baimaii *with the start of expansion estimated to be ~0.243 million years before present (MYBP) in north-east India. The populations of *An. baimaii *from north-east India had the highest haplotype and nucleotide diversity with all other populations having a subset of this diversity, likely as the result of range expansion from north-east India. The north-east Indian populations were genetically distinct from those in Bangladesh, Myanmar and Thailand, indicating that mountains, such as the Arakan mountain range between north-east India and Myanmar, are a significant barrier to gene flow. Within north-east India, there was no genetic differentiation among populations with the exception of the Central 2 population in the Barail hills area that was significantly differentiated from other populations.

**Conclusions:**

The high genetic distinctiveness of the Central 2 population in the Barail hills area of the north-east India should be confirmed and its epidemiological significance further investigated. The lack of genetic population structure in the other north-east Indian populations likely reflects large population sizes of *An. baimaii *that, historically, were able to disperse through continuous forest habitats in the north-east India. Additional markers and analytical approaches are required to determine if recent deforestation is now preventing ongoing gene flow. Until such information is acquired, *An. baimaii *in north-east India should be treated as a single unit for the implementation of vector control measures.

## Background

In the north-eastern region of India (NE India) malaria continues to be a major public health problem with 174,000 cases and 290 deaths recorded during 2010 [1]. Mosquitoes of the *Anopheles dirus, Anopheles minimus *and *Anopheles fluviatilis *complexes are the principal malaria vectors in this region. A member of the Dirus complex, *Anopheles baimaii*, (formerly *An. dirus *species D) is the main vector in forested and forest-fringe areas throughout NE India [2,3]. *Anopheles baimaii *also occurs widely in Southeast Asia, extending from NE India into the hills of Bangladesh, through Myanmar and into north-western and southern Thailand [4]. Studies using microsatellites, mitochondrial and nuclear sequence data [5] indicated that *An. baimaii *had a more confined, westerly distribution until it spread eastwards making secondary contact ~62 kyr with a closely related species, *An. dirus*, on the Thai-Myanmar border. This resulted in introgression of mtDNA from *An. baimaii *into *An. dirus *accompanied by a selective sweep of mtDNA. Both *An. baimaii *and *An. dirus *have high susceptibility to malaria parasites, high anthropophily and excellent survival rates, making them efficient malaria vectors in the sylvatic environs of Southeast Asia [6-8].

Malaria control with the existing arsenal of anti-malarials is problematic due to the emergence of multi-drug resistance in *Plasmodium falciparum*, the predominant malaria parasite in NE India [9]. Vector control is therefore a cost-effective and practical approach to reduce the burden of malaria [10]. Conventional vector control measures such as indoor residual spraying and insecticide-treated nets are effective but operationally difficult, logistically demanding and relatively less effective against exophilic and exophagic vectors such as *An. baimaii *[4]. Due to such problems, novel vector control strategies are being developed. For example, the wide scale release of genetically modified mosquitoes to render vector populations refractory to malaria parasite infection and improved sterile insect techniques are being considered for *An. gambiae *and *Aedes aegypti *[11-15], with the potential to be rolled out to other mosquito vectors if they prove successful. Whether using conventional methods or genetic-based methods of vector control, comprehensive knowledge and understanding of the biology, distribution, genetic population structure and gene flow regime of the vector species is essential for effective vector control. For example, determining genetic structure can help to understand heterogeneities in disease transmission due to genetically distinct vector populations and to predict the spread of genes of interest, such as those involved in insecticide resistance or refractoriness.

Although much is known about the geographical distribution, biology, behaviour and vectorial capacity of *An. baimaii *in NE India [2,3,16-18], its genetic diversity and population structure have been little studied within this region. Studies of genetic population structure on a broader geographical scale throughout Southeast Asia using mtDNA [19,20], microsatellites [21] and sequences of nuclear genes [5] have revealed low, but significant, structuring in *An. baimaii *and a lack of population structure in *An. dirus*. This was attributed to the greater topographical complexity in the western distribution of *An. baimaii *than the sampled areas of *An. dirus *from eastern Southeast Asia. Since NE India has a complex topography with large river basins and several different hill ranges, it is hypothesized that there would be significant genetic structuring in *An. baimaii *in this region. Whereas previous studies were unable to investigate this possibility as they used only a single population in NE India, here an extensive sampling of *An. baimaii *was conducted throughout the seven constituent states of NE India.

The preponderance of selective sweeps in mitochondrial DNA had led to criticisms for the use of this marker in studies of genetic population structure [22]. Despite such undoubted problems, mitochondrial DNA remains one of the most powerful and reliable tools for detecting population structure and inferring population history due in no small part to its high mutation rate and smaller effective population size than nuclear DNA that leads to more rapid lineage sorting and divergence between populations following their isolation [23]. Although mtDNA originating in *An. baimaii *underwent a selective sweep in *An. dirus *following introgression (see above), there is no reason to suspect that mtDNA has undergone a selective sweep in *An. baimaii *or, if it did, it has been sufficiently long ago that this marker was able to detect population structure in this species on the regional scale studied previously [20]. Therefore, mitochondrial COII gene sequences have been used to assess genetic diversity and determine genetic population structure throughout NE India and consider the implications of our findings for vector control in NE India.

## Methods

### Study area, mosquito sampling and species identification

The north-east region of India (22°04' to 29°31'N and 89°48' to 97°25'E), comprising seven administrative states *viz*. Assam, Arunachal Pradesh, Manipur, Meghalaya, Mizoram, Nagaland and Tripura, experiences a tropical climate with average annual temperature of 25°C, relative humidity of 70-80% and rainfall over 2,500 mm^3^. About 40% of the 255,128 km^2 ^area of NE India is covered with tropical forests. Topographically, the region can be broadly differentiated into the eastern Himalayas to the north, the NE Hills (Meghalaya and Mizoram- Manipur-Kachin hills) to the south, and the Brahmaputra River basin (the Brahmaputra valley) in between [24].

**Figure 1:**
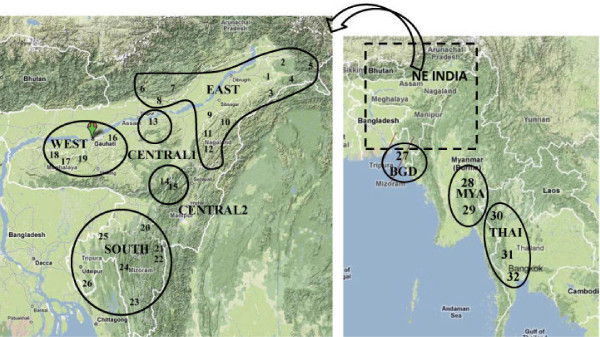
**Relief map showing collection sites of *Anopheles baimaii***. The eight population groupings (collection sites 1-32) used for *An. baimaii *are outlined by circles.

Host-seeking *An. dirus *s.l. female mosquitoes were collected in human dwellings using Center for Disease Control (CDC) light traps from 26 geo-referenced sites spread through the seven states (Figure 1, Table 1) during 2006-2009. Following morphological identification at the species complex level [25], individual mosquitoes were stored, dehydrated in beam capsules, at 4°C. Mosquitoes of the Dirus complex were subsequently identified to the species level using an allele specific polymerase chain reaction method [26].

**Table 1:** Collection sites and population groupings of *Anopheles baimaii *in NE India, Bangladesh, Myanmar and Thailand

Population Name	Name of the collection sites	**Site no**.	Coordinates (N/E)	No. of *An. baimaii* individuals sequenced
EAST	Soraipung Forest (AS)	1	N27°23' E95°34'	9
	
	Tengapani Forest (AP)	2	N27°46' E95°58	8
	
	Deomali (AP)	3	N27°09'E95°28'	9
	
	Jairampur (AP)	4	N27°20' E96°00'	4
	
	Miao (AP)	5	N27°29' E96°10'	1
	
	Kameng Bari (AP)	6	N27°04' E92°25'	2
	
	Pukke Tiger Reserve (AP)	7	N27°03' E92°36'	3
	
	Nameri National Park (AP)	8	N26°90' E92°95'	34
	
	Titabor (AS)	9	N26°36' E94°17'	1
	
	Bhandari (NL)	10	N26°17'E94°10'	8
	
	Dimapur (NL)	11	N25°54'E93°44'	1
	
	Medziphema (NL)	12	N25°50' E93°52'	12

EAST Total				92

CENTRAL 1	Kaziranga National Park (AS)	13	N26°34' E93°10'	10

CENTRAL 2	Jatinga (AS)	14	N25°11' E93°02'	4
	
	Jiribam (MN)	15	N24°47' E93°12'	3

CENTRAL2 Total				7

WEST	Kamrup (AS)	16	N26°17' E91°92'	19
	
	William Nagar (ML)	17	N25°32' E90°37'	8
	
	Tura (ML)	18	N25°51' E90°22'	1
	
	Aradonga (ML)	19	N25°55' E90°56'	2

WEST Total				30

SOUTH	Kolasib (MZ)	20	N24°14' E92°42'	2
	
	Lengpui (MZ)	21	N22°83' E92°63'	5
	
	Thenzwal (MZ)	22	N23°19' E92°45'	1
	
	Tlabung (MZ)	23	N22°53' E92°53'	8
	
	Dumpa Tiger Reserve (MZ)	24	N23°42' E92°29'	10
	
	Kailasahar (TR)	25	N24°20' E92°01'	5
	
	Sabroom (TR)	26	N23°0' E91°43'	6

SOUTH Total				37

**NE India**				**176**

BGD	Chaklapunjee	27	N21°92' E92°17'	6

MYA	Myaing	28	N19°19' E97°14'	7
	
	Kyauk	29	N16°61' E98°24'	7

MYA Total				14

THAI	Maesod	30	N16°59' E98°68'	12

	Kanchanaburi	31	N14°33' E98°99'	14
	
	Ratchaburi	32	N13°53' E99°36'	11

THAI Total				37

**Overall Total**				**233**

AS- Assam, AP- Arunachal Pradesh, NL- Nagaland, MN- Manipur, ML- Meghalaya, MZ- Mizoram, TR- Tripura, BGD- Bangladesh, MYA- Myanmar, THAI- Thailand

### Amplification and sequencing of mtDNA

A 750 base pair fragment of the mitochondrial COII gene was amplified from 176 individuals of *An. baimaii *using the AT Leu (5'ATGGCAGATTAGTGCAATGG 3') and BT Lys (5' GTTTAAGAGACCGTACTTG 3') primers [27]. The PCR reactions were performed in 50 μl volumes using a Bio-Rad thermal cycler (California, USA). Each reaction mix included 2.5 μl DNA, each primer at 0.5 μM, 3 mM MgCl_2_, 200 μM dNTP mix and 1.5 U Taq DNA polymerase in 1× PCR buffer (Roche-Scientific, Mannheim, Germany). After a preliminary denaturation step of 5 minutes at 94°C, there were 35 amplification cycles of 94°C for 45 seconds, 50°C for 45 seconds and 72°C for 60 seconds, followed by a final extension at 72°C for seven minutes. The PCR products were purified through High Pure PCR Product Purification Kits (Roche-Scientific, Mannheim, Germany) and sequenced in both directions using Big Dye Terminator V3.1 cycle sequencing kit (Applied Biosystems, USA) on a 3130 XL Genetic Analyser (Applied Biosystems, USA). Raw DNA sequences were manually trimmed and edited in BioEdit v 7.0.9 [28].

### Analysis of mtDNA variation

The edited COII sequences were aligned using Clustal W2 [29] and translated into amino acid sequences using the *Drosophila *mtDNA genetic code in MEGA version 4.0 [30]. The sequences were confirmed as mtDNA COII by comparison with the *Anopheles gambiae *sequence available in NCBI GeneBank. Information on polymorphic sites, haplotype diversity (hd), frequency of each haplotype, nucleotide diversity (π) and θs i.e. θ based on the expected number of segregating sites in a population, was extracted using DnaSP 5.0 [31]. The best model of nucleotide substitution was determined using ModelTest 3.5 [32]. The hierarchical likelihood tests and Akaike Information Criteria (AIC) [33] agreed that the HKY (Hasegawa, Kishino and Yano) model fitted the data best (Log Likelihood -1592.918; AIC 3199.837). This model was used in further analyses as appropriate. However, in Arlequin 3.1 [34] the Tamura and Nei model of evolution [35], being closest to the HKY model, was used.

### Population genetic structure

As there were many sampled sites throughout NE India, spatial analysis of molecular variance (SAMOVA) [36] was used to identify groups of populations that are geographically homogeneous and maximally differentiated from each other without assuming any prior association between populations. The algorithm identifies the optimal number of groups of populations (k) by maximizing F_CT _(the proportion of total genetic variance due to differences among groups of populations) and minimizing F_SC _(genetic differentiation among populations within groups). The SAMOVA analysis was run using 10,000 simulated annealing steps from each of 100 sets of initial starting conditions for k = 2-24.

Analysis of molecular variance (AMOVA) for the optimal groups was carried out in Arlequin 3.1. AMOVA takes into account the number of molecular differences between haplotypes in an analysis-of-variance framework equivalent to F-statistics. Pairwise F_ST _values were calculated in Arlequin 3.1 and their significance was tested by 1,000 permutations. To test if the pattern of population differentiation followed an isolation-by-distance model [37,38], a Mantel test was conducted in Arlequin 3.1. This used a matrix of linearized pair-wise genetic distances, estimated by F_ST _[F_ST_/1-F_ST_] with corresponding geographic distances among populations calculated using the software Geographic Distance Matrix Generator version 1.2.3 [39].

Spatial structuring within NE India was assessed using the Spatial Analysis of Shared Alleles (SAShA) method [40]. This method is based on the premise that if lineages are evolving locally then the same alleles are expected to co-occur in the same location more often than expected by chance. SAShA detects structure by testing if alleles are more geographically restricted than expected under panmixia by testing if the observed mean of geographical distances between every pair of identical alleles is less than the expected mean assuming panmixia. The expected mean is estimated from the mean of the geographical distances between all pairs of alleles.

A rarefaction method, as commonly used in ecological studies to estimate species diversity, was modified according to Colwell *et al *[41] to estimate the total number of haplotypes present in the NE Indian populations. In this modification, populations (localities) were treated as "samples" and haplotypes treated as "species". EstimateS 8.2.0 [42] was used to generate haplotype accumulation curves under the assumption of equal sampling effort across populations although it was noted that the studied sample sizes differed between localities. Accuracy of the method will also be reduced if populations vary significantly in haplotype diversity. Despite these considerations, the asymptote of the haplotype accumulation curve provides a reasonable enough estimate of the total number of haplotypes for the purposes here.

### Genealogical relationships and population history of mtDNA haplotypes

A haplotype network was constructed using the median joining (MJ) algorithm [43] in NETWORK 4.5. This method was used as it performs well when there are un-sampled internal node haplotypes in the network [44] as occurs here due to the high diversity and high number of haplotypes present. The frequency distribution of the number of pair-wise differences between haplotypes, known as a mismatch distribution, is smooth and unimodal in a recently expanded population [45]. To test if *An. baimaii *in NE India has undergone expansion, a mismatch distribution was calculated in Arlequin 3.1 and compared with the expectations of the sudden expansion model. The time of expansion was estimated under the sudden expansion model assuming the mutation rate of 1.15 × 10^-8^/site/year as used by Morgan *et al *[5] and a generation time of 0.1 years reported for Anophelines (Maharaj, 2003) [46].

To test the null model of neutral mutation and constant population size Tajima's D [47] and Fu's Fs test [48] were used. Tajima's D tests if the estimates of θ based on the number of segregating sites and θ based on the average number of pair-wise nucleotide differences are same as expected under the null model. Fu's Fs test [48] assesses if the number of observed alleles is in excess of that expected given the observed level of genetic diversity under the null model. All these neutrality tests were conducted in DnaSP 5.0 and their significance assessed by 10,000 coalescent simulations.

## Results

### Sequence variation

A total of 176 individuals of *An. baimaii *from 26 sites in NE India were sequenced for 636 bp of the COII gene. No pseudogenes were present as evidenced by the absence of stop codons, the prevalence of synonymous substitutions, low pair-wise divergence and unambiguous electropherograms. All haplotype sequences from NE India have been deposited in GenBank (accession numbers GeneBank:HQ202747 to HQ202801 and GeneBank:HQ650203 to HQ650217). To this dataset 57 COII sequences from one population in Bangladesh (n = 6), two populations in Myanmar (n = 14)and three populations in Thailand (n = 37) were added from a previous study [20] to enable comparison on a regional scale (Table 1). In the total dataset 83 mutations were found, 71synonymous and 12 non-synonymous with 47 parsimony informative sites. Overall haplotype diversity was high with a total of 103 haplotypes, but it was highest in NE India (89 haplotypes from 176 sequences) with a haplotype diversity of 0.95 (± 0.012) across all NE Indian populations (Table 2).

**Table 2:** Summary statistics of *Anopheles baimaii *population groups

Population Group	N	Haplotype distribution*	S	H	hd ± SD	π ± SD	**θ**_**S**_ ± SD	Tajima's D	Fu's Fs
EAST	92	1(2),2, 3, **4(2)**, 5, 6, 7, **8(13), 9**, 10, **11(5), 12(5)**, 13, **14, 15, 16(2)**, 17, 18, 19, **20 **(2), 21,22(2), 23, 24, **25(2)**, 26, 27, **28, 29**, 30, **31**, 32, 33, 34, 35, 36, 37, 38, 39, 40, **41**(2), 42, 43(2), **44(3), 45**, 46, **47**(2), 48, 49, **50**, 51, 52, 53(2), 54, **55**, 56, 57	54	57	0.972 0.002	0.0064 0.0035	0.017 0.005	-2.057***	-25.93***

CENTRAL 1	10	**8**(5), **20, 25**,58, 59, 60	8	6	0.778 0.045	0.0031 0.0022	0.0044 0.0023	-1.399	-10.84 ***

CENTRAL 2	7	**8, 9, 15, 16, 26**, 61(2)	11	6	0.952 0.076	0.0076 0.0048	0.0071 0.0037	-0.016	-3.29 **

WEST	30	**8**(9), **9**(2), **12(4), 14, 29, 31, 44, 45, 47**, 62(2), 63, 64, 65, 66, 67, 68, 69	24	17	0.899 0.009	0.0053 0.0031	0.0095 0.0034	-1.682*	-26.20 ***

SOUTH	37	**4, 8**(8), **9, 11**(2), **12, 16, 25(2), 41**, 70, 71, 72, 73, 74, 75, 76, 77, 78, 79, 80, 81, 82, 83, 84, 85, 86, **87**, 88, 89	40	28	0.955 0.006	0.0062 0.0035	0.0150 0.0049	-2.183***	-25.97 ***

**All NE Indian Populations**			74	89	0.950 0.012	0.0057 0.0003	0.0091 0.0037	-2.277**	-16.09 ***

BGD	6	**8, 12**(3), **55**, 90	5	4	0.800 0.096	0.0030 0.0023	0.0034 0.0021	-0.826	-4.56***

MYA	14	**8**(3), **12**(4), **50**, 91(2), 92(2), 93, 94	7	7	0.879 0.027	0.0026 0.0018	0.0035 0.0018	-1.019	-20.38***

THAI	37	**8**(17), **11, 12**(5), **25, 28, 87**, 95, 96, 97, 98(3), 99, 100, 101, 102, 103	16	15	0.776 0.006	0.0026 0.0017	0.0060 0.0021	-1.910**	-27.60***

*Haplotypes in bold occur in more than one population group and the number in parentheses indicates the frequency of a haplotype at a particular site.

N = number of individuals sequenced; S = number of segregating sites; H = number of haplotypes; hd = haplotype diversity; π = nucleotide diversity per site; θs = Genetic diversity estimated from segregating sites; SD = Standard Deviation

Level of significance is indicated by: * = < 0.05, ** = < 0.01, *** = < 0.001

### Population genetic structure

In the SAMOVA analysis, the *F*_CT _values decreased from k = 2 to k = 24 indicating an overall lack of genetically distinct population groups. However, the highest value of *F*_CT _was for k = 2 (*F*_CT _= 0.22; p < 0.05), in which population 14 (Jatinga, Assam) was separated from all other populations, indicating that population 14 is substantially genetically distinct. There appears to be some genetic distinctiveness of some of the NE Indian populations as they separated one by one from k = 3 to k = 7 respectively (populations 15, 7, 1, 4,2). The two most genetically distinct populations, 14 and 15, are adjacent to one another and not differentiated from each other (pair-wise *F*_ST _0.06, p < 0.4) but there was no discernable spatial pattern among the other genetically distinct populations. From k = 8 onwards *F*_SC _was negative indicating no further discernable differentiation among populations within the large group of remaining populations. Up to k = 16 the six populations from Bangladesh, Myanmar and Thailand always grouped together further highlighting the distinction between them and the NE Indian populations.

Since SAMOVA was unable to detect obvious population clusters, in order to detect any subtle levels of genetic differentiation using larger sample sizes, samples from all 32 locations were grouped based on their geographical proximity into eight population groups: five population groups from NE India and one population each from Bangladesh, Myanmar and Thailand (Figure 1). Analysis of molecular variance (AMOVA) using these groupings revealed that the majority of variation (96.8%) occurred within populations. Pair-wise F_ST _among the eight population groups revealed some population structuring at a large spatial scale with the Bangladesh, Myanmar and Thai populations being significantly differentiated from two, three and four of the five NE Indian populations, respectively (Table 3). This corresponds to the signal of isolation by distance also seen on this large spatial scale; the Mantel test using all 32 populations was significant (r = 0.236, p < 0.02). A Mantel test using only the NE Indian populations found no pattern of isolation by distance (r = 0.062, p > 0.2). There was also no significant differentiation among the NE Indian populations with the sole exception of the Central 2 population group (from the Barail hills area) which comprised populations 14 and 15, detected by SAMOVA to be the two most genetically distinct sites. The Central 2 group was highly differentiated from the Bangladesh, Myanmar and Thailand population groups (Table 3; e.g. F_ST _= 0.35 for the Thailand/Central 2 comparison) and differentiated from all other NE Indian groups with the exception of the West NE Indian population group (p = 0.07).

**Table 3:** Genetic differentiation (F_ST_) among *Anopheles baimaii *populations

	EAST	CENTRAL1	CENTRAL 2	WEST	SOUTH	BGD	MYA
CENTRAL 1	-0.009						

CENTRAL 2	0.071*	0.175**					

WEST	-0.004	0.013	0.074				

SOUTH	-0.007	-0.010	0.065*	-0.002			

BGD	0.032	0.146**	0.180*	0.026	0.060		

MYA	0.024	0.063*	0.267***	0.031	0.050**	0.014	

THAI	0.055***	0.043	0.351***	0.069***	0.078***	0.071	0.002

Level of significance is indicated by: * = p < 0.05, ** = p < 0.01, *** = p < 0.001

Sample Sizes: EAST (n = 92), CENTRAL1 (n = 10), CENTRAL2 (n = 7), WEST (n = 30), SOUTH (n = 37), BGD (n = 6), MYA (n = 14) and THAI (n = 37)

When lineage diversity is high, as here, it can be hard to detect differentiation since populations tend to comprise alleles from genetically divergent lineages. Since there was some indication from the SAMOVA of genetic distinctiveness within the NE Indian region the Spatial Analysis of Shared Alleles (SAShA) method [40] was used to assess if there was more subtle evidence for population structuring. The SAShA analysis found that the spatial arrangement of COII haplotypes was equi-distributed and not statistically different from the expectation under panmixia (observed mean 297.01 km, expected mean 279.76 km, p = 0.22). However, haplotype accumulation curves (data not shown) estimated that ~250 haplotypes (95% CI, 160-410) are expected to be present, compared to 89 observed in the NE India, i.e. the majority of haplotypes in northeast India were unsampled. Consequently the SAShA approach has little power to detect structure if it exists since this method relies on being able to resample the same haplotype multiple times. All the methods were therefore unable to detect evidence for genetic structuring throughout the NE India with the exception of populations 14 and 15 in the Central 2 group as noted above.

### Genetic diversity

The genetic diversity and θ estimates for the eight populations of *An. baimaii *are presented in Table 2. The NE Indian populations had higher genetic diversity than the Bangladesh, Myanmar and Thailand populations of *An. baimaii *with overall values of haplotype diversity (hd) of 0.950 ± 0.012, nucleotide diversity of 0.0057 ± 0.0003 and θs of 0.0091 ± 0.0037. Within NE India the genetically distinct Central 2 population group was similarly genetically diverse to most other groupings but the Central 1 population had markedly lower values of the genetic diversity indices.

### Demographic expansion

**Figure 2:**
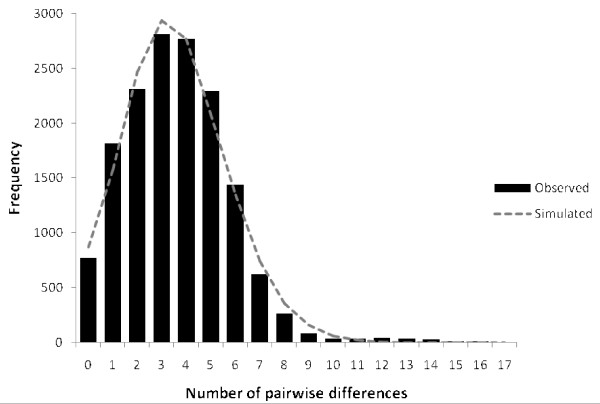
**Mismatch distribution among haplotypes of *Anopheles baimaii *from the NE Indian populations**.

The neutrality test statistics (Table 2) were found negative for all the eight populations with some populations having significant values. However, Fu's Fs was found to be significant for all populations. Among the NE Indian populations, both Tajima's D and Fu's Fs statistics were significantly negative (Tajima's D -2.2772, p < 0.01; Fu's Fs -16.085, p < 0.001). These negative values indicate population growth with the very high significance of the Fu's Fs test reflecting the power of this statistic [48]. A unimodal mismatch distribution (Figure 2) was also consistent with population expansion as a model of expansion could not be rejected for the observed data (*p *= 0.27 and SSD 0.026; *p *= 0.30). It should be noted that all these tests do not distinguish demographic expansion from a selective sweep of mtDNA, which is an expansion of a population of molecules carrying a favoured mutation. The start time of population expansion under the sudden expansion model was estimated using the same mutation rate as in earlier studies to enable a direct comparison with previous estimates for a single NE Indian population of *An. baimaii *[5,20]. However, since there is no calibrated rate for *Anopheles*, the estimated expansion time should be considered as very approximate. It was found that *An. baimaii *in the NE India expanded approximately 0.243 million years before present (MYBP) (95% CI 0.11-0.39) is in broad agreement with the earlier estimates from a single population of about 0.30 MYBP.

### Genealogy

**Figure 3:**
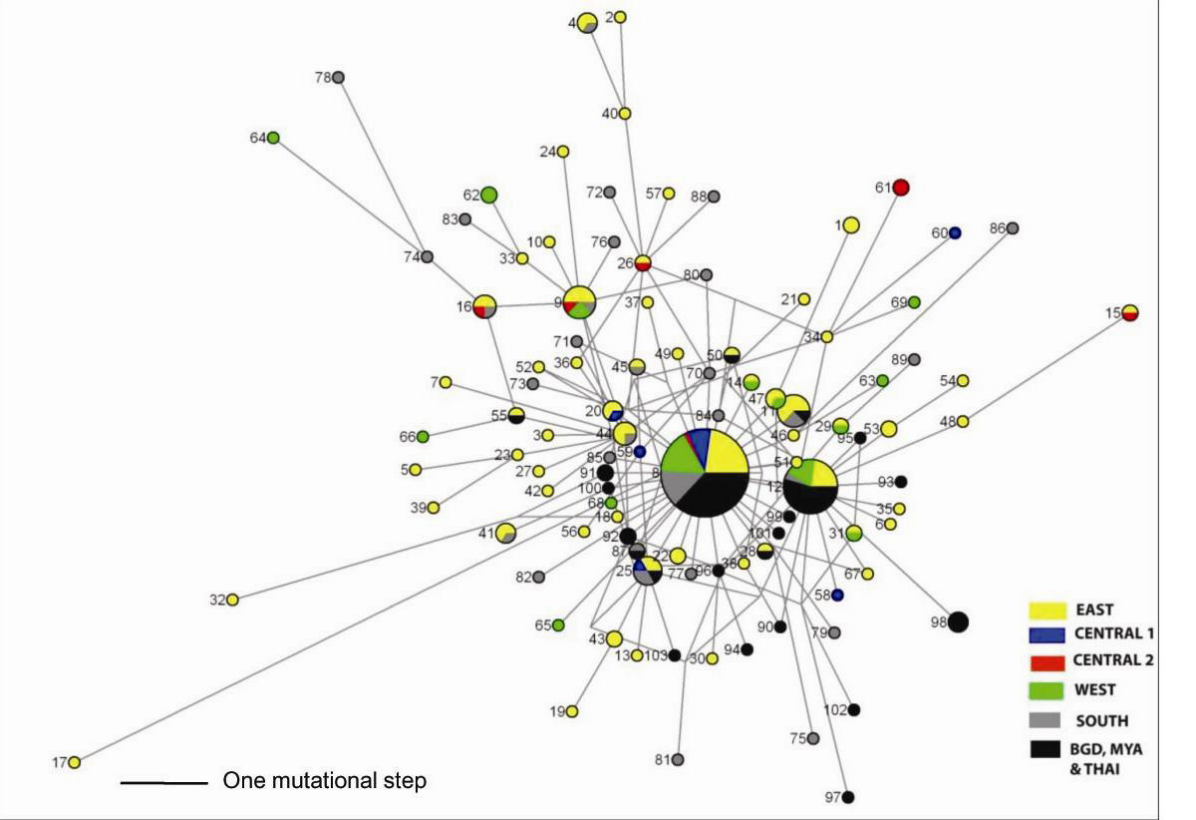
**Median joining haplotype network of 103 COII haplotypes of *Anopheles baimaii *showing the relationships between the haplotypes from the different NE Indian populations and the Bangladesh, Myanmar and Thailand populations**. The colour and the size of the circles represent the geographic origin and frequency of each haplotype, respectively. The length of the lines connecting haplotypes is proportional to the number of mutational differences separating the haplotypes.

The median joining haplotype network has an overall star-like topology (Figure 3). There are two very high frequency, widespread haplotypes (H8 and H12) at the centre of the network, surrounded by many low frequency derived haplotypes, a pattern typically indicative of recent population expansion or a recent selective sweep [49]. The longer lineages were comprised almost exclusively of the NE Indian haplotypes with no pronounced clustering according to population of origin, indicating that the NE Indian populations have similar population histories (Figure 3). Even the highly divergent haplotype (H17) from the East population, that was separated from the core haplotype H8 by 10 mutations, was confirmed as that of *An. baimaii *using the PCR-based identification method (see Methods) of Walton *et al *[26] based on the ribosomal DNA ITS2 region. The presence of two closely related but rare haplotypes (H9 and H16) in the Central 2 population explain the distinctiveness of this population group detected by SAMOVA and pair-wise AMOVA. Duplicate copies of a haplotype restricted to a particular population group i.e. H1, H22, H43, H61 and H62, were also suggestive of some restrictions to gene flow. However, these were insufficient to give a positive result for the SAShA analysis. The majority of haplotypes from Bangladesh, Myanmar and Thailand were one of the two core haplotypes and the remaining haplotypes from these locations generally differed by only one or two mutational steps, indicating a very different demographic history from the NE Indian populations (Figure 3).

## Discussion

This is the first extensive study of *An. baimaii *in NE India with sampling spanning all seven states. In agreement with earlier studies it was found that *An. baimaii *in NE India had substantially higher molecular diversity (i.e. θs and nucleotide diversity) than populations in Bangladesh, Thailand and Myanmar. This is in accord with NE India being the origin of all extant populations of this species [5]. A further contributing factor to this high genetic diversity may be that much of NE India has acted as a refugium for forest species during Pleistocene glacial cycles when forests were reduced and fragmented across Southeast Asia due to lower temperatures and increased aridity [[50] and refs therein]. The signal of population expansion dating to the late Pleistocene is consistent with this, as during interglacials forests and forest-dependent taxa would expand from refugial areas. The impacts of glacial cycles may have been lower in NE India due to its very high rainfall that results from its unique geography: mountain ranges surrounding valleys such as those of the Brahmaputra and Barak rivers that force the monsoons to rise resulting in very high rainfall [51]. The thesis of NE India as a Pleistocene refugial area is in keeping with the high species diversity of the area [52,53] as high species diversity is often due to long term environmental stability [54].

The wide sampling coverage of our study was able to reveal that not only is there exceptionally high mtDNA diversity of *An. baimaii *throughout NE India but, as shown by the haplotype network (Figure 3), all the mtDNA haplotypes elsewhere in this species are either a limited subset of those present throughout NE India or are only very recently derived from those in NE India. The multiple, distinctive mtDNA lineages within NE India indicate that there has not been a recent selective sweep in NE India itself. Rather, the data are more consistent with the bottlenecking of mtDNA outside of NE India being due to a demographic bottleneck. This is consistent with the inference from combined nuclear and mtDNA data made by Morgan *et al *[5], that populations of *An. baimaii *spread from NE India through Myanmar to the Thailand border where they made secondary contact with *An. dirus*. These therefore further support the inference in Morgan *et al *[5] that the selective sweep occurs on introgression into *An. dirus*.

This marked bottlenecking of mtDNA on moving out of NE India results in the observed genetic differentiation of *An. baimaii *from the populations in Bangladesh, Myanmar and Thailand and indicates the presence of substantial geographical barriers to gene flow from/into NE India. The distribution of *An. baimaii *[3], was found to be widely present in the foothills and hills of the NE Indian plains. Therefore its absence closer to the Myanmar border (based on this study's sampling in the middle of Mizoram state and in Manipur state) can be attributed to the high elevation and relatively colder temperature of these areas. Since the Arakan mountain range (including the Naga hills and Chin hills) and the Patkai range (including the Lushai (Mizo) hills) that divide NE India from Myanmar average about 2,000-3,000 metres, these mountain ranges are therefore likely to be a substantial barrier to dispersal between NE India and Myanmar (Figure 1). As the land between NE India and Bangladesh is of lower elevation it is not clear why the Bangladesh population also appears to be bottlenecked (low genetic diversity and limited distribution of haplotypes across the haplotype network) but we cannot exclude an artifactual effect due to the low sample size from Bangladesh (n = 6).

There is some correlation between geographic and genetic distance on a large spatial scale (i.e. across the countries) but not on a smaller spatial scale (i.e. within NE India) consistent with isolation by distance on a regional scale. However, as there are relatively few sample sites outside of NE India, the possibility that the apparent isolation by distance is due, at least in part, to successive geographical barriers to gene flow cannot be excluded. For example, the Shan Plateau and Tenasserim mountain range with altitudes of up to 1,000 metres that separate Thailand and Myanmar are a likely barrier to dispersal. This is concordant with the observation of no isolation by distance observed within NE India despite the considerable distances involved and the lack of high mountains between the sample sites. Similarly, a previous study using nuclear loci [5] found no differentiation between north and South Thailand, which are also distant but not separated by mountains. Further sampling on a regional geographical scale is required to fully distinguish the alternatives of large-scale isolation by distance and montane barriers to gene flow.

Within NE India, the population groups sampled appeared to be somewhat geographically isolated, with a lack of suitable intervening habitat (i.e. primary or minimally disturbed evergreen forests). Despite this, very little genetic structure was apparent, counter to the original hypothesis stated. Instead, the very high genetic diversity and lack of structuring (with the exception of the Central 2 population group from the Barail hills area) indicate large effective population sizes and genetic homogeneity. Given the low sample size of the Central 2 population group (seven individuals) further sample collection and genetic analysis should be done to confirm its genetic distinctiveness. However, the high levels of significance found here indicate that Central 2 really is genetically distinct. In addition, there were also some other indications of isolation among the NE Indian populations: the splitting off of several NE Indian populations from k = 2 to k = 7; and the low genetic diversity of the Central 1 population that indicates it has undergone a genetic bottleneck and became isolated.

Forests in NE India were until recently contiguous with lowland semi-evergreen forests throughout the Bramaputra valley merging into sub-Himalayan light alluvial semi-evergreen and secondary wet mixed forests in the hilly regions [55]. Large scale fragmentation of these forests occurred only relatively recently, within the last 150-200 years, due to human population increase and economic growth [52,56]. Therefore it can likely be considered that the general signal of genetic homogeneity reflects the historical genetic connectivity of *An. baimaii *populations throughout NE India and that the slight signals of genetic isolation result from recent human-mediated forest fragmentation.

Control of *An. baimaii *and malaria transmitted by it is a challenging task in areas of its influence, due to its predominant exophagic and exclusive exophilic behaviour, along with its efficient vectorial capacity. Lack of genetic structure and presence of genetic homogeneity, as observed here, may be the likely reasons for its universally efficient vectorial status in this part of India. Use of insecticide treated nets (ITNs) in combination with repellents have been found very effective in controlling malaria transmitted by *An. baimaii *in the north-east India [57]. At present *An. baimaii *in this region is susceptible to DDT (used in indoor residual spraying) and pyrthroids (used in ITNs) [58], but in view of the possibility of gene flow among populations of this mosquito, close monitoring of insecticide susceptibility status in NE India is warranted.

## Conclusions

*Anopheles baimaii *populations from NE India are more genetically diverse and genetically distinct from *An. baimaii *in the rest of Southeast Asia; the latter likely as a result of high elevations being a barrier to gene flow. Therefore it should not be assumed that biological features recorded for *An. baimaii *in one region of Southeast Asia necessarily apply to *An. baimaii *in another region. For example, it was noted that some populations in Myanmar differ with respect to larval habitat choice, breeding in wells in villages rather than in groundwater in the forest [59,60]. Within NE India, biological attributes of *An. baimaii *such as breeding, biting and resting behaviours, response to insecticides, and malaria transmission, appear to be uniform throughout much of the region [2,16,61], consistent with the overall lack of population genetic structuring observed here. If the genetic distinctiveness of the Central 2 population is confirmed by further genetic analysis on larger sample sizes (currently n = 7), detailed bionomical studies of *An. baimaii *are needed in the Barail hill range of the NE India to determine if the genetic distinctiveness of the Central 2 population corresponds to any changes in biological traits that affect malaria transmission or vector control. It is important to note that in the Central 2 population areas *An. baimaii *occurs sympatrically with the closely related, recently reported species × of the *An. dirus *complex [3]. Further studies should therefore include a thorough evaluation of the epidemiological significance of both species.

The findings of this study highlight the difficulty of detecting recent structuring in populations when there are very high population sizes and high genetic diversity, as multiple divergent lineages occur in all locations and there will be a substantial time lag before haplotype frequencies vary between populations. The extent of ongoing (rather than historical) gene flow is needed for vector control but determining this will be challenging. It will require the use of multiple markers combined with innovative analytical approaches to provide the power needed to distinguish ongoing gene flow from the potential confounding effects of demographic history.

## Competing interests

The authors declare that they have no competing interests.

## Authors' contributions

This study forms part of the PhD thesis work of DKS. DKS collected mosquitoes, performed the data analysis and drafted the manuscript. DKS and SMO'L performed molecular analyses including PCR and DNA sequencing of the COII gene fragment. AP helped in the field collections, data analysis and in preparation of manuscript. DRB, NPS and SS helped in mosquito collections. KD and KB performed the molecular identification work. PKM and GUA helped in drafting of the manuscript. AP, JM and CW participated in the design of the study, data analysis, draft of manuscript, general supervision of the research group and fund acquisitions. All authors read and approved the final documents.
